# Carbon-doped BN nanosheets for metal-free photoredox catalysis

**DOI:** 10.1038/ncomms8698

**Published:** 2015-07-10

**Authors:** Caijin Huang, Cheng Chen, Mingwen Zhang, Lihua Lin, Xinxin Ye, Sen Lin, Markus Antonietti, Xinchen Wang

**Affiliations:** 1 State Key Laboratory of Photocatalysis on Energy and Environment, College of Chemistry, Fuzhou University, Fuzhou 350002, China.; 2 Department of Colloid Chemistry, Max Planck Institute of Colloids and Interfaces, Potsdam 14476, Germany.

## Abstract

The generation of sustainable and stable semiconductors for solar energy conversion by photoredox catalysis, for example, light-induced water splitting and carbon dioxide reduction, is a key challenge of modern materials chemistry. Here we present a simple synthesis of a ternary semiconductor, boron carbon nitride, and show that it can catalyse hydrogen or oxygen evolution from water as well as carbon dioxide reduction under visible light illumination. The ternary B–C–N alloy features a delocalized two-dimensional electron system with *sp*^2^ carbon incorporated in the *h*-BN lattice where the bandgap can be adjusted by the amount of incorporated carbon to produce unique functions. Such sustainable photocatalysts made of lightweight elements facilitate the innovative construction of photoredox cascades to utilize solar energy for chemical conversion.

## Abstract (web summary)

Metal-free semiconductors with appropriate bandgaps create photocatalytic routes to water splitting and CO_2_ reduction. Here the authors dope hexagonal boron nitride nanosheets with carbon via a simple method to synthesize a ternary B–C–N alloy capable of performing just this function.

## Graphical abstract

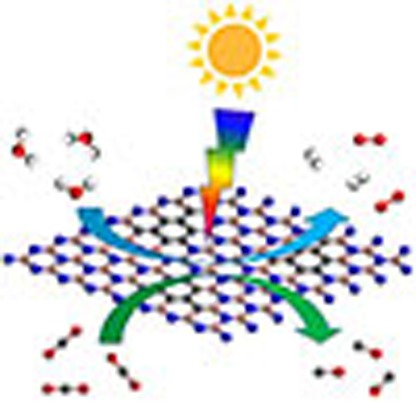

The use of abundant sunlight to split water into H_2_ and O_2_ has been considered as the simplest pathway to make clean fuels that can be converted to electricity, for example, in H_2_-O_2_ fuel cells^1^,^2^. Since the report on photocatalytic water splitting in 1972 (ref. 3), a quantum yield of ∼56% has been achieved for NiO/NaTaO_3_-La at 270 nm for overall water splitting^4^, while for visible light systems a highest quantum yield of ∼5% at 420 nm was reported for RhCrO/ZnGaON^5^. These studied promise solar fuel production by photocatalysis. Further progress, however, relies on breakthroughs in the development of sustainable photocatalysts and co-catalysts, classically based on metals^6^,^7^ (for example, metal oxides, (oxy)nitride and (oxy)sulfides) for light harvesting, together with noble metal co-catalysts (for example, Pt and RuO_2_) for driving the two half reactions, water reduction and water oxidation. The two half reactions can be later combined to construct two-photon (Z-scheme) photocatalytic systems to achieve overall water splitting in a cascade fashion^8^, as inspired by the photosynthetic reactions in plants.

Recently, a class of metal-free photocatalysts has been emerged, including elemental boron^9^, sulfur^10^ and phosphor^11^, as well as the binary carbon nitride^12^ and boron carbide^13^. These papers indicate that this construction of stable photocatalysts from lightweight and abundant elements is possible and opens up new opportunities for photochemistry. The two-dimensional (2D) character of their electron systems gives further access to partly ‘exotic' physical properties, as described for graphene^14^ and layered hexagonal BN (*h*-BN)^15^,^16^,^17^. Graphene, the single atomic layer of carbons expresses a zero bandgap, whereas the *h*-BN possesses a wide bandgap (∼5.5 eV). Both materials are therefore, as photocatalyst, not suitable, but intermediates of these layered materials (called ternary BCN compounds) could constitute the desired medium-bandgap semiconductors where bandgap and absolute energy levels can be adjusted by chemical variations^18^,^19^.

Here we present a facile synthesis method to produce a ternary structure of BCN nanosheets that has a functionality to catalyse hydrogen and oxygen evolution from water as well as CO_2_ reduction under visible light illumination.

## Results

### Theoretical calculations

**Figure 1:**
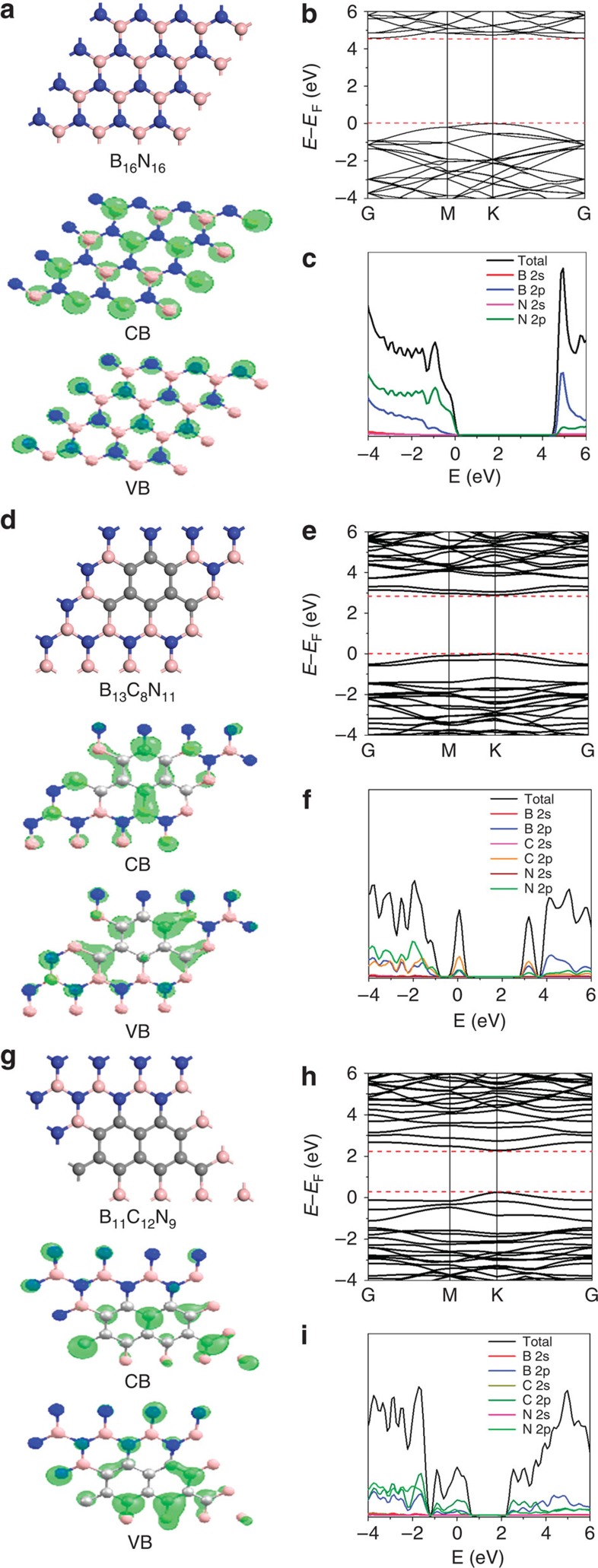
The plane-wave DFT calculations of electronic structure of *h*-BN and *h*-BCN. The optimized structure of B_16_N_16_ (**a**) B_13_C_8_N_11_ (**d**) and B_11_C_12_N_9_ (**g**) with the corresponding valence band (VB)/conduction band (CB), calculated energy band (**b**,**e**,**h**) and corresponding total and ion-decomposed electronic density of states (**c**,**f**,**i**).

From density functional theory (DFT) calculations, pure *h*-BN possesses an indirect bandgap of 4.56 eV (Fig. 1a–c), consistent with the previous theoretical values^20^. This is about 1.00 eV smaller than the experimental value^21^, due to the well-known limitation of the DFT within GGA method. As shown in Fig. 1g for a ‘B_11_C_12_N_9_'-compound, the gap is significantly reduced from 4.56 to 2.00 eV. Different from that of pure *h*-BN (Fig. 1c), the partial density of states indicates that the valence band and conduction band edges of B_11_C_12_N_9_ are mainly composed of the C *2p* orbitals (Fig. 1i). Contrary to the localized valence band top of pure *h*-BN, the valence band top states of B_11_C_12_N_9_ are more delocalized as displayed in Fig. 1h. Calculation results on the B–C–N alloy with a different composition of ‘B_13_C_8_N_11_' are displayed in Fig. 1e,f, and their computational optical absorption was displayed in Supplementary Fig. 1. Similar results on band structure were attained, but with an enlarged gap of 2.90 eV due to the reduced carbon content in *h*-BN. Clearly, the doping of aromatic carbon into the *h*-BN lattice narrows its bandgap, making it possible to induce visible light catalysis^20^,^22^.

### Preparation of BCN-*x* samples

Doping of graphene with boron and nitrogen is generally performed to open up its bandgap^23^, but it is still challenging to keep this process sufficiently controlled. The adjustment of the carbon doping within *h*-BN^18^ seems to us the more easy access to BCN-based materials with high yield in a controllable manner. Herein, we develop such a carbon-doping strategy by using a pyrolysis method. In brief, different amounts of glucose were mixed with boron oxide and urea. The mixture was put into a tube furnace in an ammonia atmosphere for 5 h at 1,250 °C (see Methods section for details). The obtained samples were named as BCN-*x*, where *x* is the glucose weight in per cent of boron oxide.

### Characterization of BCN-*x* samples

**Figure 2:**
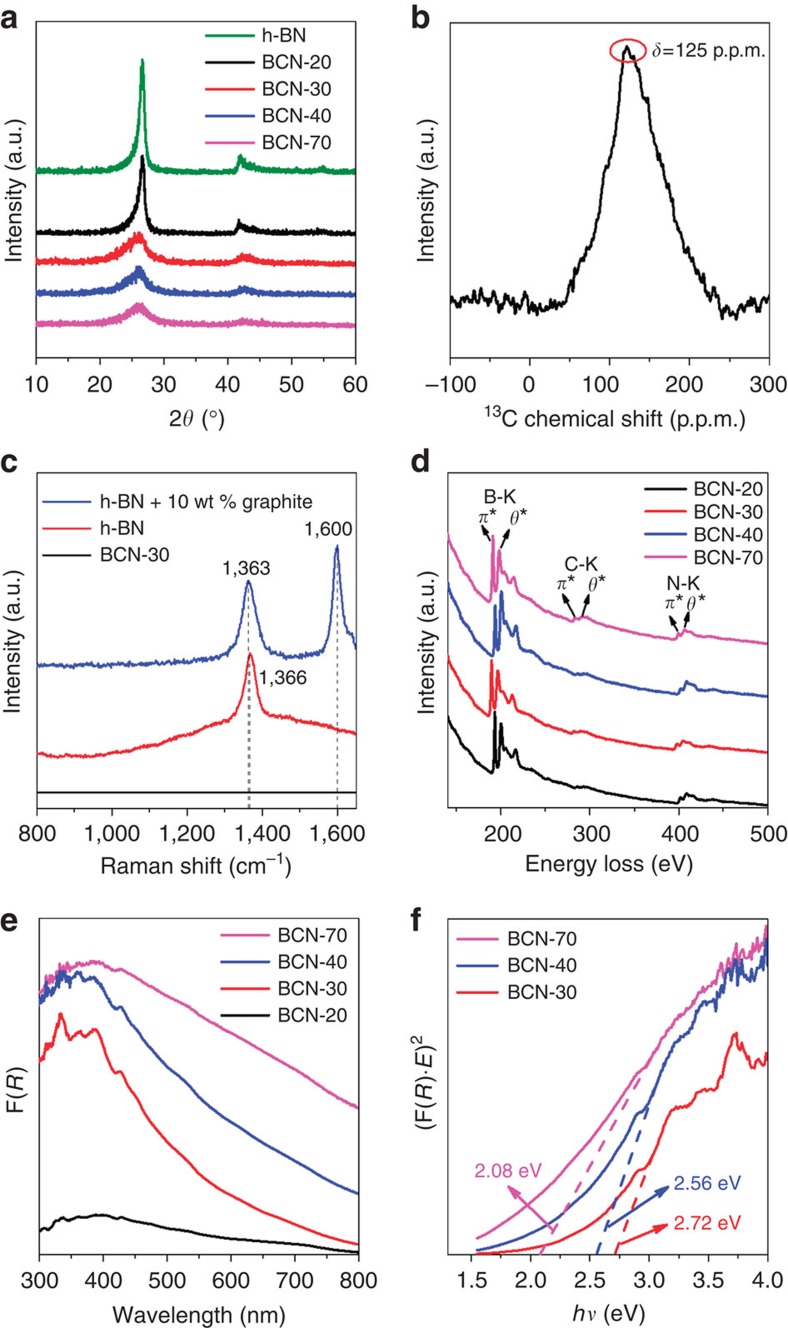
Structure and optical characterization of *h*-BCN. (**a**) Powder XRD patterns of *h*-BCN synthesized with different amount of glucose, (**b**) solid-state ^13^C NMR spectrum of BCN-30, (**c**) Raman spectra of the *h*-BN, BCN-30 and the physical mixture of *h*-BN and graphite, (**d**) EELS spectra of BCN-*x* sample, (**e**) ultraviolet–visible diffuse reflectance spectra (UV–vis DRS) of the BCN-*x* samples and (**f**) bandgap determination of the BCN-*x* samples from the (F(R)·E)^n^ versus *E* plots. According to the result of theoretical calculation of *h*-BCN (Fig. 1g,k), the band structure of *h*-BCN is the direct gaps, so the ‘*n*' is equal to 2.

Figure 2a shows the X-ray diffraction (XRD) patterns of the prepared samples with different amounts of glucose. Without glucose, the synthesis yielded *h*-BN showing two characteristic XRD peaks at ∼26° and ∼43°, attributable to the (002) and (100) planes of the graphitic structure of *h*-BN, respectively^24^. With glucose, all samples featured XRD peaks similar to those of *h*-BN, while increasing amounts of glucose broadens the peaks^25^, accompanied by a slight shift of the peak to lower angles. This indicates the doping of carbon in the *h*-BN lattice, forming ternary B–C–N alloys.

The chemical structure was further characterized with Fourier transform infrared (FT-IR; Supplementary Fig. 2). Two bands observed at 1,380 and 780 cm^−1^ correspond to the in-plan B–N transverse stretching vibration and the out-of-plan B–N–B bending vibration, respectively^26^. We failed to detect the C–N bonds, which typically overlap with B–N bands around 1,100–1,300 cm^−1^. ^13^C solid-state NMR was therefore performed to show the presence of carbon (Fig. 2b). Interestingly, the resonance appears around 125 p.p.m., similar to that of reduced graphene oxide/graphite^27^, which corresponds to *sp*^2^ carbons with an electron density very similar to graphite. This means that in this BCN alloy the influence of the electronegativity of N and electropositivity of B balance each other out, with the carbon as such being only slightly more electron rich than in graphite. The incorporation of carbon in the *h*-BN layers smears out Raman signals of *h*-BN due to the distortion of the layer symmetry^28^ (Fig. 2c). Note that the Raman peaks of *h*-BN remain for the physical mixture of *h*-BN and graphite, measured as a reference.

Supplementary Fig. 3 is the X-ray photoelectron spectroscopic (XPS) spectra of the BCN product. The main peak of B*1s* at 190.4 eV is attributed to B atoms surrounded by N atoms^29^. The subpeaks at 189.9 and 192.3 eV are due to the B–C band and unreacted B–O, the latter potentially being a surface impurity^25^. The C*1s* signal at 284.6 eV is due to the graphitic carbon (C=C), while the peaks at 285.7 and 283.5 eV are assigned to C–N bonds and C–B bonds, respectively^29^. The corresponding N-signals for N–B or N–C bands are also observed and located at 397.9 and 398.6 eV (ref. 29). The small subpeak at 400.5 eV reflects N–H bonds, again presumably needed for edge termination and reflecting the reductive ammonia atmosphere. Elemental analysis by XPS (Supplementary Table 1) revealed that the carbon content is gradually increasing with increasing amounts of glucose. Thus, XPS analysis very clearly supports the incorporation of carbon in the *h*-BN.

Figure 2d displays the electron energy loss spectroscopy (EELS) of the *h*-BCN samples, showing the K-edges of B, C and N. The B- and N-signal of BCN coincide with those of *h*-BN, reflecting a similar incorporation in layered *sp*^2^ domains^18^,^23^. The sharp doublet C–K (π*) and C–K (θ*) evidence a rather perfect *sp*^2^-bonding neighbourhood also for the carbon positions. In addition, the carbon doping actually commutatively affects the corresponding B–K, N–K and C–K energies. All data indicate that B, C and N are in a commonly *sp*^2^-hybridized, 2D-conjugated electron system, rather than a physical mixture.

**Figure 3:**
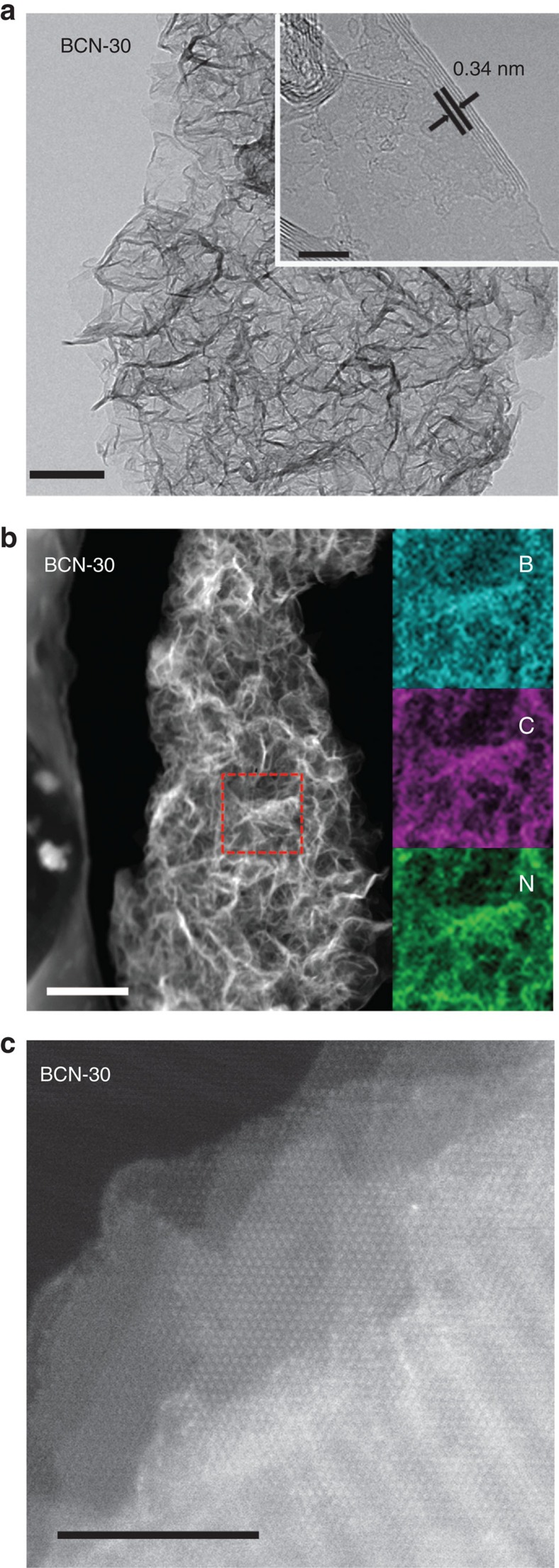
Surface morphology of BCN-30. (**a**) HRTEM image of the BCN-30 sample. Scale bar, 100 nm, inset scale bars, 5 nm. (**b**) Typical TEM dark-field image of BCN-30 sample and the elemental mapping images of B, C and N of the enlargement of selected-area in the picture. Scale bar, 300 nm. (**c**) High-resolution STEM of the BCN-30 sample along (002) facet. Scale bar, 5 nm.

Figure 3 depicts transmission electron microscopy (TEM) and STEM images of the BCN-30, and the scanning electron microscopy and energy-dispersive X-ray spectroscopy analyses were presented in Supplementary Fig. 4. A flake-like morphology similar to few-layer-graphene gives evidence that folded BCN layers constitute the structure. High-resolution TEM (inset of Fig. 3a) allows the observation of an interlayer crystal lattice spacing of 3.40 *Å.* The nanosheets are composed of ∼10 stacked layers and have 3–4 nm overall thickness. Such 2D systems with thicknesses below 10 nm are particularly interesting for optoelectrics as the exciton and charge diffusion length is generally in that size range^30^. The selected-area electron diffraction (inset of Supplementary Fig. 5) also suggests that the in-planar order of the sample is polycrystalline, which might reflect that the carbon forms a solid solution or constitutes grain boundaries of BN nanodomains. Element mapping of BCN-30 (Fig. 3b) gave a uniform distribution of B, C and N throughout the whole selected area, proving homogeneity of the ternary BCN alloys. This is also proven by the STEM image that clearly displays homogeneous mixing of atoms in the (002) facet (Fig. 3c), without finding grain boundaries. The ternary BCN is rather stable up to 800 °C in the air, even (Supplementary Fig. 6).

The optical absorption of the samples is shown in Fig. 2e. Carbon-free *h*-BN made as such is a typical insulator with a bandgap of about 5.6 eV (refs 16, 21). As shown in Fig. 2f, the optical absorption edge of BCN alloys gradually red-shifts as the amount of carbon increased. From the plot of the transformed Kubelka–Munk function versus light energy, a bandgap of 2.72 eV was estimated for BCN-30. The long absorption tails in the visible however indicates the presence of intraband impurity transitions, potentially located at the surface. Further optimization of the synthesis of high-quality *h*-BCN alloys is certainly a valuable option. Nevertheless, for a proof-of-concept advance, the optical properties are sufficient to generate electron–hole pairs on *h*-BCN as confirmed by photoelectrochemical (Supplementary Fig. 7) and electron paramagnetic resonance characterizations (Supplementary Fig. 8a,b). The band positions of the *h*-BCN is estimated to straddle the water redox potentials, which are however tuneable by the amount of carbon doped (Supplementary Fig. 9). Therefore, we could apply these new semiconductors for the first time for visible light photocatalysis.

## Discussion

**Figure 4:**
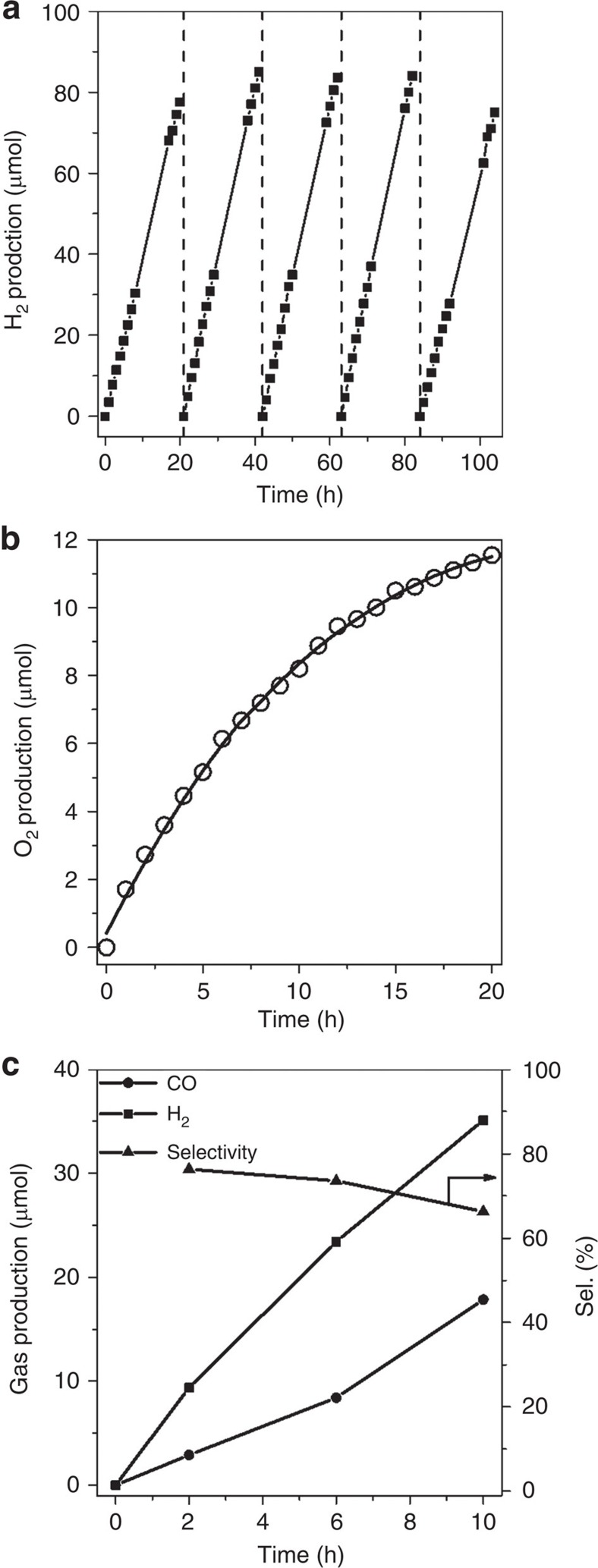
Photocatalytic performance of BCN-30. (**a**) Stable hydrogen evolution from water by BCN-30 under visible light. The reaction was continued for 104 h, with evacuation every 20 h (dashed line). (**b**) Time courses of oxygen production from water by Ni-Co LDHs/BCN-30 under visible light illumination (*λ*>420 nm). (**c**) Time conversion plot for photocatalytic CO_2_ reduction. The amount of CO and H_2_ produced from the CO_2_ conversion system as a function of reaction time under visible light illumination (*λ*>420 nm). Selectivity=*n*CO/*n*(CO+H_2_) × 100%.

The photocatalytic performance of BCN samples was assessed by a H_2_ evolution assay. H_2_ evolution under light was found, while in dark the samples are inactive. The best sample is BCN-30, while hydrogen evolution rate gradually decreases as the carbon content further increases (Supplementary Fig. 10). Excessive amounts of carbon presumably diminish the size of ordered domains and weaken the semiconductor properties, as confirmed by the decrease in the charge-carrier lifetime with excessive carbon doped (Supplementary Fig. 11). It is however a remarkable observation that the BCN alloys could photocatalyse hydrogen generation even without using noble metals as a cocatalyst and even with visible light. Under those conditions, the samples present a quite stable activity (Fig. 4a), but a deactivation was observed when the materials was illuminated with ultraviolet light (Supplementary Fig. 12a).

We also modified BCN with Pt for H_2_ evolution, but observed only comparable H_2_ production in the range of 0.5–3 wt % Pt (Supplementary Fig. 13). This indicates that the surface of BCN alloys are already rather active, and one can speculate about a pro forma hydride elimination from the boron sites (corresponds to a reversible hydrogenation of a –N=B-bond) in the catalytic cycle. However, the stability of BCN under strong ultraviolet irradiation is remarkably increased by adding Pt (Supplementary Fig. 12b). We can speculate that the Pt is avoiding the over-reduction of the –N=B-structure and the consecutive reductive disintegration of the material. The overall production of hydrogen gas amounts to 4,570 μmol in these two-cycle reactions, exceeding the amount of *h*-BCN (1,342 μmol) and Pt (5.12 μmol). After the reaction, the structure of BCN-30 was stable as reflected by the XRD, FTIR and TEM examinations of the used photocatalyst (Supplementary Fig. 14). The apparent quantum efficiency of BCN-30 was calculated to be 0.54% at 405 nm (Supplementary Table 2) by using equation (1). We also compared the performance of BCN-30 with other photocatalysts, and results show that the activity of BCN-30 is higher than that of TiO_2_ (P25) and g-C_3_N_4_ under visible light (Supplementary Table 3). It is noted that there is not clear relationship between the specific surface area and hydrogen evolution rate of BCN samples (Supplementary Table 4). This is due to the fact that in most solid–liquid phase photocatalysis the reaction rate is basically limited by charge separation instead of mass transfer.

The H_2_ generation from water is pleasing, but it constitutes only the minor half of the problem of water splitting. The other half-reaction, the oxygen evolution reaction, is particularly challenging because it requires the promotion of four redox processes over a narrow potential range, the coupling of multiple proton and electron transfers, and the formation of an oxygen–oxygen bond. As a first trial to promote oxygen evolution from water by BCN, we choose RuO_2_ and IrO_3_, and Ni-Co layered double hydroxides (Ni-Co LDHs) as cocatalysts^31^. Results revealed that Ni-Co LDHs is the best cocatalyst (Supplementary Fig. 15), potentially due to intimate interface contacts between the two layered structures. As shown in Fig. 4b, the Ni-Co LDHs/BCN-30 catalyst liberated 11 μmol O_2_ in 20 h reaction with visible light, and with ultraviolet light the experiment gave 35 μmol O_2_ (Supplementary Fig. 16).

These results indicate that the band positions of *h*-BCN are correctly positioned for water splitting, that is, the Ni-Co LDH is able to take up the photogenerated holes and evolves O_2_. The decrease in activity with reaction time is primarily due to the deposition of metallic silver at the catalyst surface, which blocks light absorption and obstructs active sites^12^. Further optimization of the O_2_ evolution system is thus certainly needed as the found activity is only moderate.

Having a very reductive photoelectron however allows to address the direct photochemical conversion of CO_2_ (ref. 32). We therefore expanded in a last experiment the application of *h*-BCN to the photocatalytic reduction of CO_2_ to CO (Fig. 4c) using visible light (*λ*>420 nm). On irradiation for 2 h, the system evolved CO (9.3 μmol) and H_2_ (2.9 μmol). A prolonged operation of the photochemical system gave a gradual increase in both CO and H_2_ evolutions in a linear fashion. Isotopic experiments confirmed the carbon source of the produced ^13^CO is ^13^CO_2_ (Supplementary Fig. 17).

In summary, we introduced here a novel and simple way of carbon doping of *h*-BN nanosheets to generate ternary BCN alloys as sustainable and stable visible light photocatalysts. Water splitting in the elements as well as CO_2_ reduction was proven to be possible. This new photocatalyst features a 2D electron system and tuneable bulk and surface properties, and further structural optimizations can be expected that may facilitate innovations and applications in the fields from artificial photosynthesis to graphene-like semiconductors, as well as organocatalysis.

## Methods

### Synthesis of BCN-*x*

Typically, boron oxide (2 g), urea (4 g) and a certain amount of glucose were grinded fully with an agate mortar. After that, mixed precursor was put into a horizontal tube furnace. Before heating up, it costs nearly 30 min to expel the whole oxygen in the tube and the sample was then heated to 1,250 °C for 5 h. The obtained products were washed with 0.1 M HCl in hot water. The resulting final sample was denoted as BCN-*x*, where *x* (20, 30, 40, 70) is the percentage weight content of glucose to boron oxide. When the glucose was omitted, the synthesized sample was named *h*-BN.

### Synthesis of Ni-Co LDHs/BCN-30

Co(NO)_2_·6H_2_O (0.2 g), Ni(NO_3_)_2_·6H_2_O (0.1 g) and NH_4_NO_3_ (0.04 g) were dissolved in H_2_O (3.5 ml) and 30 wt% ammonia (1.5 ml) to form clear solution, named as Ni-Co LDHs. 200 mg BCN-30 powder was dispersed in water (20 ml), which was then subject to ultrasonic treatment for 10 min to promote the dispersion of BCN-30 in the solution. A certain amount of the Ni-Co LDHs solution (400 μl) was added to the mixture. After stirring for 3 h, the mixture was washed by filtering with distilled water and dried at 343 K to obtain Ni-Co LDHs/BCN-30.

### Characterization

Powder XRD patterns were collected on Bruker D8 Advance diffractometer with Cu-K1 radiation (*λ*=1.5406 Å). Data were collected with a rate of 0.02°/ 2θ in the range of 10 to 60°. The FT-IR spectra were obtained on a Nicolet 670 FT-IR spectrometer with KBr as the diluents. To get smooth spectra, the final results were registered after accumulation of 108 scans and a resolution of 4 cm^−1^. XPS measurements were performed on an ESCALAB 250 (Thermo Scientific, USA) by using a monochromatized Al Kα line source (200 W). Solid-state ^13^C MAS NMR experiments were conducted on a spectrometer (Bruker Avance III-400WB) equipped with a 4-mm ZrO_2_ rotor probe operating at a 5 kHz spinning rate for ^13^C nuclei. The direct ^13^C-polarization spectrum was acquired with a 90° pulse of 5 μs for a pulse repetition delay of 2 s for 2,048 scans. The ^13^C spectrum was referenced to tetramethylsilane at 0 p.p.m. using adamantane as an external reference. Raman spectroscopic measurements were performed on a Renishaw in Via Raman System 1000 with a 532 nm Nd:YAG excitation source at room temperature. The UV/Vis picture was recorded on a Cary 500 Scan Spectrophotometer (Varian, USA). Here BaSO_4_ was used as a reflectance standard in the ultraviolet–visible diffuse reflectance experiment. Nitrogen adsorption–desorption isotherms were collected at 77 K using a Micromeritics ASAP 2020 surface area and porosity analyser. Thermogravimetric analysis was performed on TG209 (NETZSCH Co.). Photoluminescence spectra were recorded on an Edinburgh FI/FSTCSPC 920 spectrophotometer. The morphology of the sample and energy-dispersive X-ray spectroscopy were investigated by Hitachi S4800 field emission scanning electron microscopy. TEM was operated by Tecnai20 FEG microscope. The elemental mapping, and selected-area electron diffraction were also collected on the TEM machine. High-resolution STEM and EELS investigation were carried out on a JEM-ARM200CF microscope (JEOL, Tokyo, Japan) with double CS correctors for the condenser lens and objective lens at an operating voltage of 80 kV. HAADF images were acquired at acceptance angles of 70–150 mrad. Photoelectrochemical analysis was conducted with a BAS Epsilon Electrochemical System in a three electrode cell, using an Ag/AgCl electrode (3 M KCl) as the reference electrode and a Pt plate as the counter electrode. The working electrode was prepared by spreading with catalyst slurry (5 mg ml^−1^ in DMF) on indium-tin oxide. After air drying, the working electrode was put into a 0.2-M Na_2_SO_4_ aqueous solution (pH=6.8). Electron paramagnetic resonance analysis was conducted with A Bruker A300 spectrometer, with the following settings: centre field, 3512.48 G; microwave frequency, 9.86 GHz; and power, 6.35 mW.

### Theoretical calculation

The plane-wave DFT calculations were carried out by using the Vienna *ab initio* simulation package with the gradient-corrected PW91 exchange-correction function. For valence electrons, a plane-wave basis set was adopted with an energy cutoff of 400 eV and the ionic cores were described with the projector augmented-wave method. The optimized lattice parameter for a *h*-BN monolayer was calculated to be 2.49 Å, which is in good agreement with the experimental result (2.54 Å)^33^. A 4 × 4 supercell *h*-BN containing 32 atoms of *h*-BN is constructed as a substrate. For the optimization of all the relaxation atoms, 3 × 3 × 1 Monkhorst–Pack *k*-point grids were used to sample the Brillouin zone, which were tested to be converged, whereas for electronic properties calculations, the Brilliouin zone was sampled by 9 × 9 × 1 *k*-points. In addition, to avoid the interlayer interactions, a vacuum spacing in the *z* direction was set to be 14 Å. Here the possible structure of the BCN was constructed based on our experimental section. At present, there is a great controversy of the BCN structure. Some argue that the BN and C present independent just like a carbon island or BN island while other think the C is a part of the BN, which means that there is a C–B or C–N bond in the compound. In our synthetic section, it possessed 5 h to reaction under high temperature. Maybe in the early reaction section, the C, B, N can group freely, but later the compound will reform to a lower free energy compound. Previous theory and experiment results have already testified that the bonding energy of C–C and B–N bonds is comparatively higher than that of other hetero bonds present in the ternary system, consequently, the hybrid systems will be domain-segregated into two different regions, one with C-rich domain and other with BN-enriched domain, to gain thermodynamic stability. Fortunately, our results are coincided with this common sense. So, we build the possible structures like above.

### Photocatalytic reaction for water reduction and oxidation

Reactions were experimented with a Pyrex top irradiation reactor connected to a glass closed gas-circulation system. Photoreduction to H_2_ and photooxidation to O_2_ were performed separately in aqueous solutions containing triethanolamine or silver nitrate as sacrificial reagents, respectively. To study the H_2_ production ability, 50 mg catalyst was dispersed into 100 ml aqueous containing triethanolamine solution (10 vol%). In the case of deposition of Pt, an appropriate amount of H_2_PtCl_6_ aqueous was added to the reactant solution before irradiation. Water photooxidation was carried out by dispersing the catalyst (50 mg) in 100 ml aqueous solution containing silver nitrate (0.01 M) as the electron acceptor and La_2_O_3_ (0.2 g) as the pH buffer agent (pH 8–9). The reactant solution was evacuated several times to remove air completely before irradiation under a 300 W xenon lamp with an appropriate cutoff filter. The temperature of the solution was maintained at 12 °C by a flow of cooling water during the reaction. The evolved gases were analysed by a SHIMADZU GC-8 A gas chromatograph with the thermal conductive detector, a 5-Å molecular sieve column and using Argon as the carrier gas.

The apparent quantum efficiency (AQE) for H_2_ evolution was performed in the same experiment set-up but with a 405 nm diode laser as incident light source. The irradiation area was 7.92 cm^2^. The total intensity of irradiation was determined by averaging 40 points of the irradiation area and the average intensity was estimated to be 4.24 mW cm^−2^ (ILT 950 spectroradiometer). The AQE was calculated as follow:


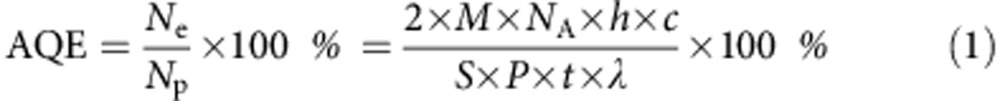


Where, *N*_p_ is the total incident photons, *N*_e_ is the total reactive electrons, *M* is the amount of H_2_ molecules, *N*_A_ is Avogadro constant, *h* is the Planck constant, *c* is the speed of light, *S* is the irradiation area, *P* is the intensity of irradiation light, *t* is the photoreaction time and *λ* is the wavelength of the monochromatic light.

### Photocatalytic reduction of CO_2_

The test was conducted at atmospheric pressure of CO_2_ in a two neck flask (50 ml) at 30 °C as controlled by water with a constant temperature. The photocatalytic CO_2_ reduction reaction was carried out by dispersing 50 mg catalyst in a solution containing solvent of 2 ml H_2_O and 4 ml acetonitrile, 1 ml triethanolamine, 1 μmol CoCl_2_, 20 mg 2, 2-bipyridine. This mixture system was subjected to vacuum degassing and backfilling with pure CO_2_ gas (1 bar). Then the reaction flask was performed under an irradiation of 300 W xenon lamp with 420 nm cutoff filter. The produced gases (CO and H_2_) were detected using a gas chromatography equipped with a packed molecular sieve column (TDX-1 mesh 42/10). Argon was used as the carrier gas.

## Additional information

**How to cite this article:** Huang, C. *et al*. Carbon-doped BN nanosheets for metal-free photoredox catalysis. *Nat. Commun.* 6:7698 doi: 10.1038/ncomms8698 (2015).

## Supplementary Material

Supplementary Information Supplementary Figures 1-17 and Supplementary Tables 1-4
