# Supplementary material for: Carbon-doped BN nanosheets for metal-free photoredox catalysis
Source: Nat Commun. 2015 Jul 10;6:7698. doi: 10.1038/ncomms8698 (PMC4510690; doi:10.1038/ncomms8698)
Supplement: Supplementary Information — Supplementary Figures 1-17 and Supplementary Tables 1-4 [file ncomms8698-s1.pdf]

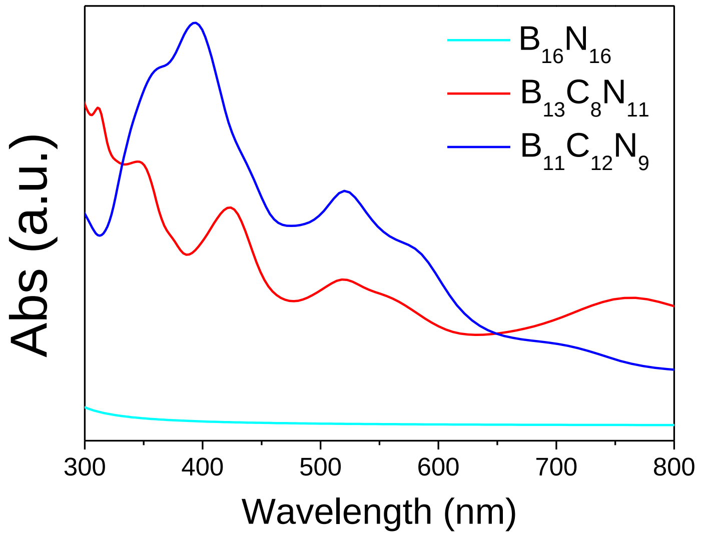

Supplementary Figure 1. Computational optical absorption of the proposed BCN structures in Fig. 1. The result is in reasonable agreement with the experimental observation in Fig. 2e.

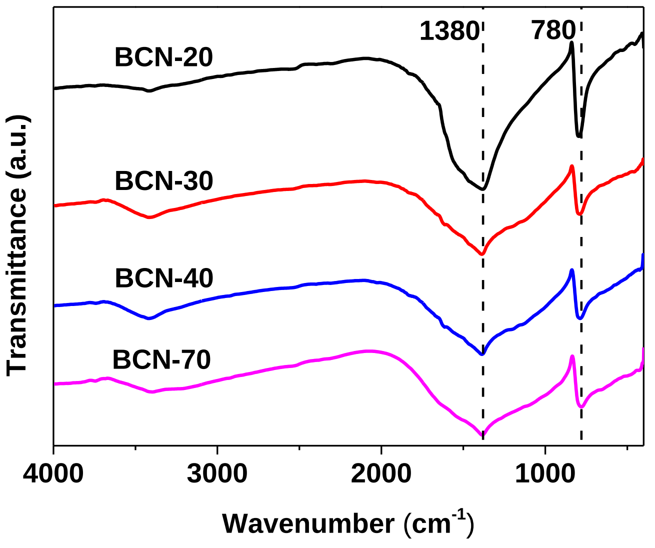

Supplementary Figure 2. FT-IR spectra of BCN-x samples.

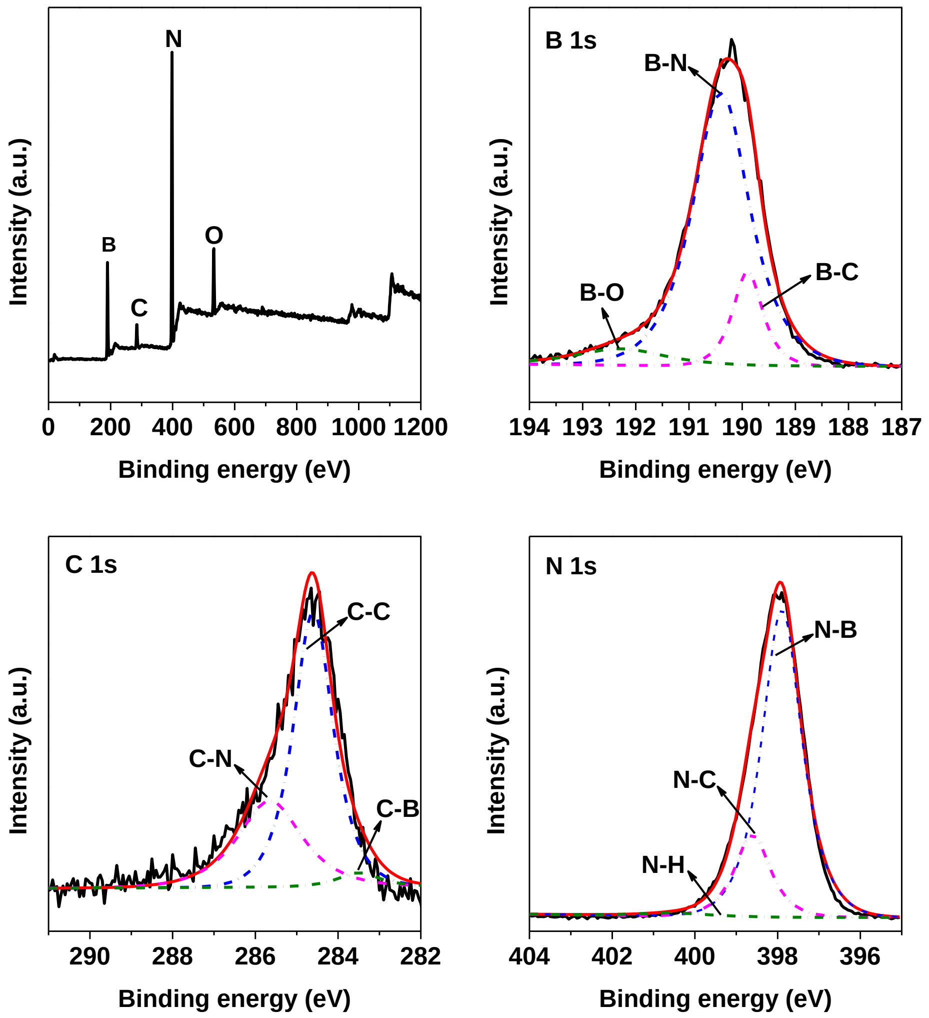

Supplementary Figure 3. XPS survey and high-resolved spectra of BCN-30 sample.

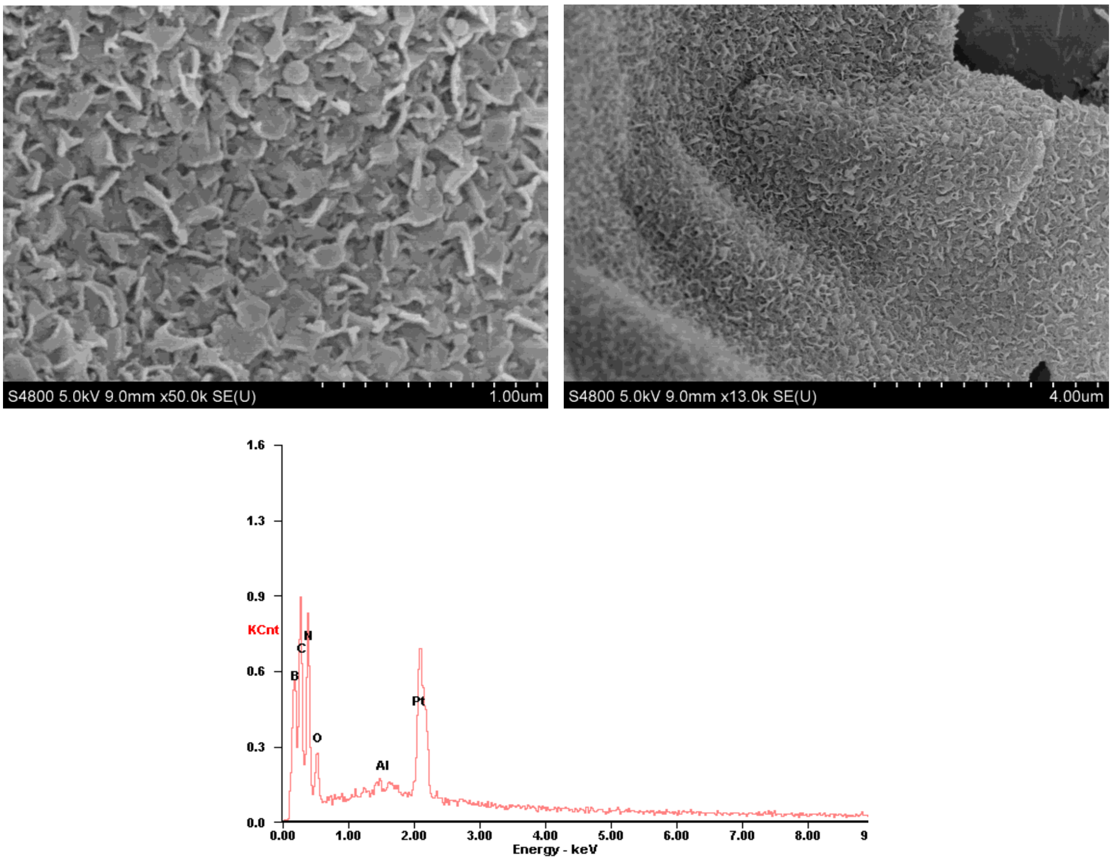

Supplementary Figure 4. The macroscopic morphology of the BCN-30 sample by SEM analysis, together with the energy-dispersive X-ray (EDX) spectroscopy analyses. The oxygen is coming from the un-reacted B_2O_3 residues. The Pt is coming from the conductive coatings on the BCN-30 during the sample preparation for the SEM measurement and the Al is coming from the conductive substrate to load BCN-30 sample.

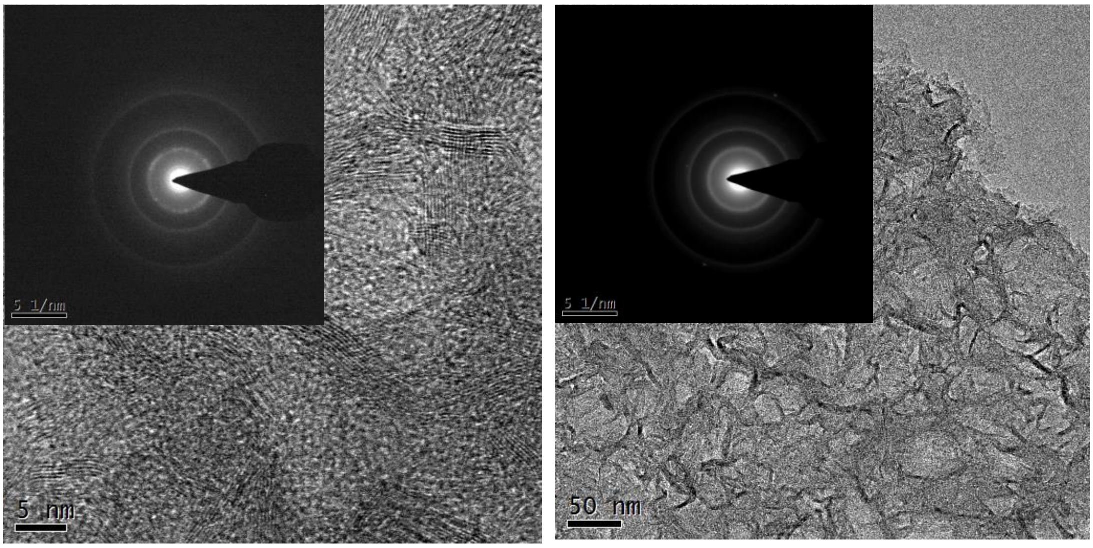

Supplementary Figure 5. TEM images and selected-area electron diffraction of BCN-30 sample.

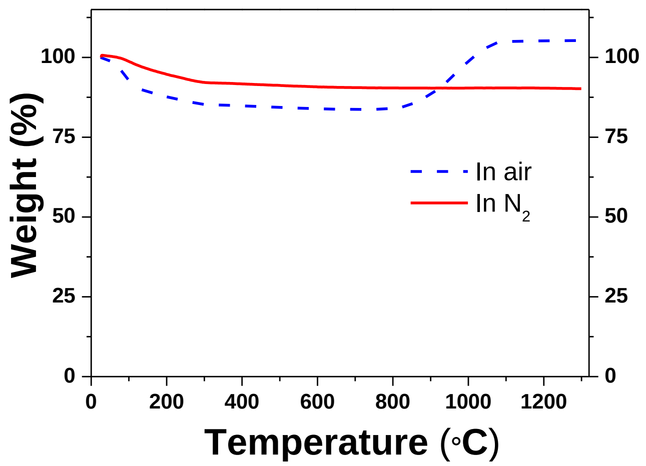

Supplementary Figure 6. The thermogravimetric analysis of the BCN-30 sample. After the desorption of water below ca. 200 °C, there is not significantly weight loss up to 1250 °C, revealing the high thermal stability of the sample in N_2 atmosphere. When carrying the analysis in air, the material is stable up to ca. 800 °C, above which the increase in the weight is related to the oxidation of BCN to produce corresponding oxides, like B_2O_3.

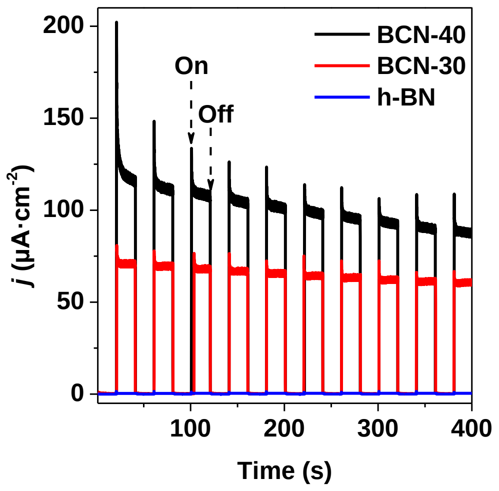

Supplementary Figure 7. The photo-current generation of different samples with light illumination. The main process of the photcatalytic water splitting is the photo-generated and separated electrons and holes of the interface of the sample BCN and water molecular. To explore the real course, photoelectrochemistry setup may be the authoritative and powerful tool to monitor this process and show a believable mechanism to explore the photocatalytic reaction. Obviously, the working electrodes could be photo-induced electro-hole pairs under the light illumination. Increasing the carbon amount, the photo-current is higher. Because the carbon doped into the BCN, the electron delocalizability of the sample is changed, which is similar to the theoretical calculation.

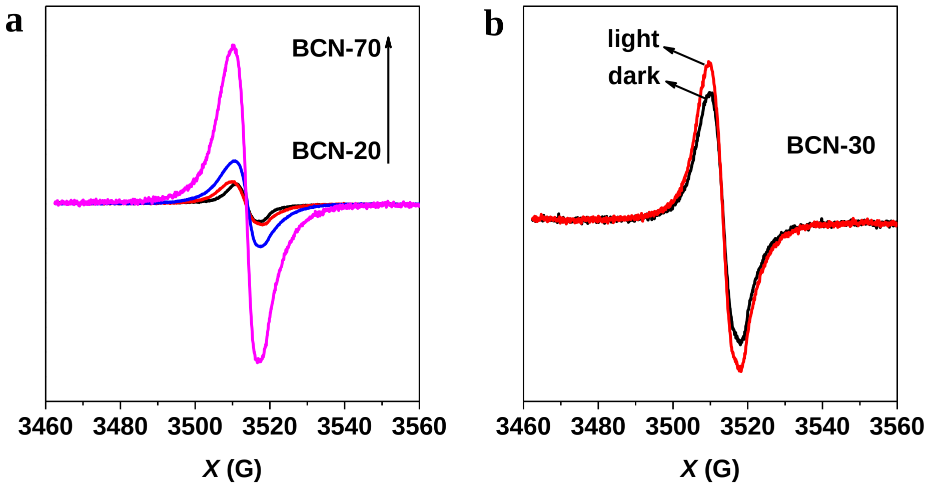

Supplementary Figure 8. Room-temperature EPR spectra of h-BCN. a, EPR spectra of the BCN-x samples in the dark. **b,** EPR spectra of BCN-30 in visible-light irradiation (λ> 420 nm, red line) and dark (black line). The electron paramagnetic resonance (EPR) spectrum of the BCN shows a slightly asymmetric signal line at a *g* value of 2.0032, manifesting a small *g* anisotropy of a uniform electron species. It is reported that the unpaired electron on carbon atoms in aromatic systems have the same *g* value. In addition, the EPR signals are enhanced upon light irradiation, indicating that the semiconductors by excitation are capable of generating electron-hole pairs. The EPR characterization gives a stand proof that indeed BCN alloys have been synthesized.

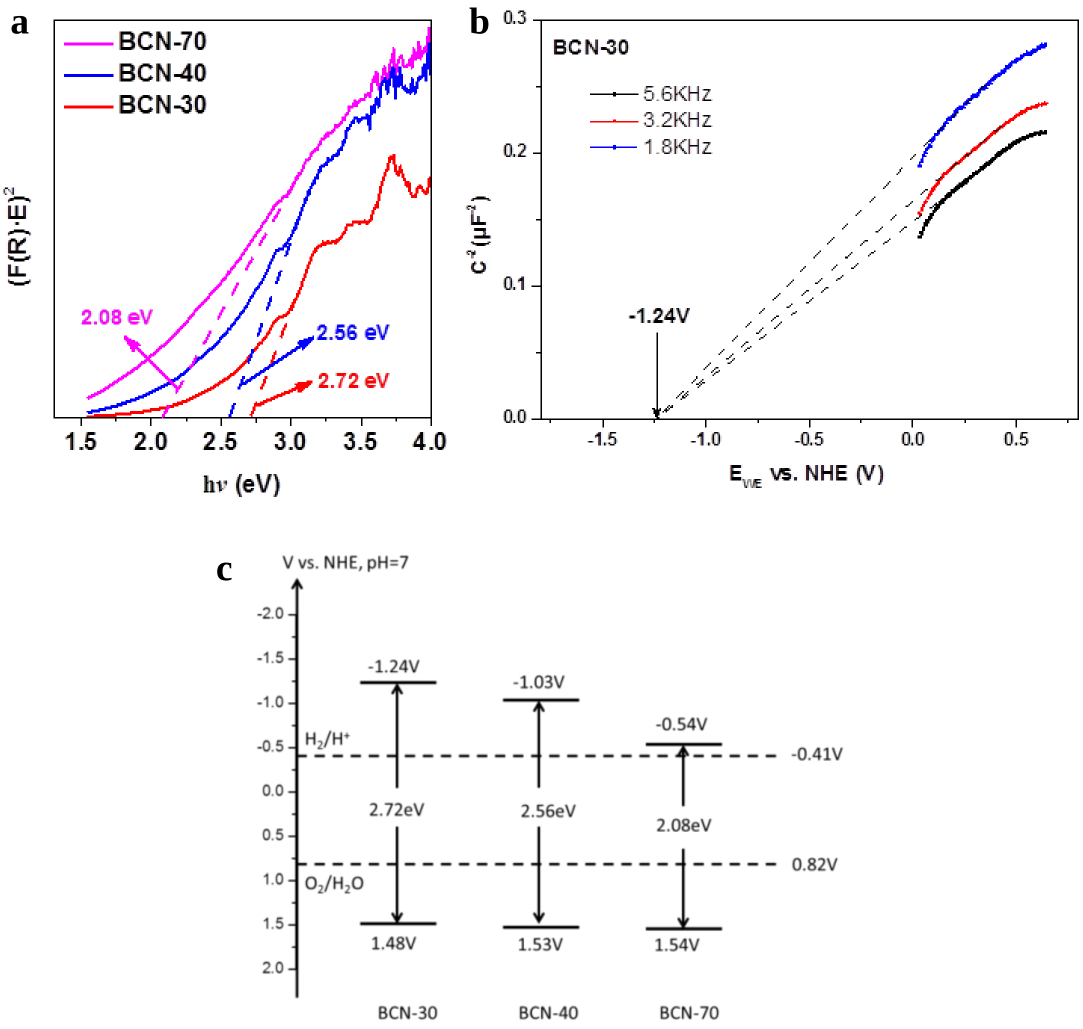

Supplementary Figure 9. The band structure of the samples. **a,** The Tauc plots derived from the ultraviolet–visible diffuse reflectance spectrum (Fig. 2e) of BCN-30, BCN-40 and BCN-70 to determine their band gap energy. **b,** The determination of the conduction band minimum (CBM) of BCN-x (in the case of BCN-30 sample as an example) by Mott-Schottky method. **c,** The determined band structure of the samples with respect to Normal hydrogen electrode (NHE), together with the reduction level for H^+ to H_2 as well as the oxidation potential of H_2O to O_2.

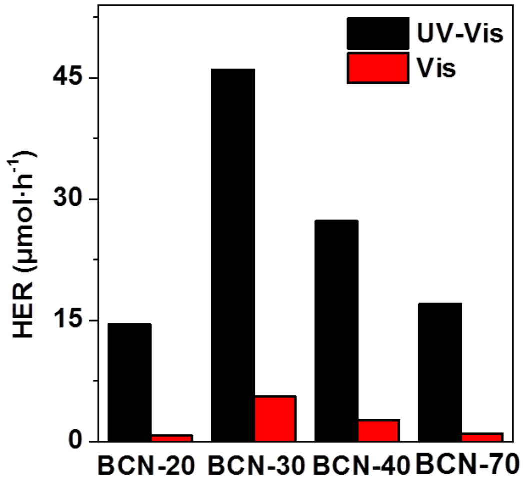

Supplementary Figure 10. The hydrogen evolution rate (HER) over 1.0 wt% Pt-loaded BCN-x photocatalysts.

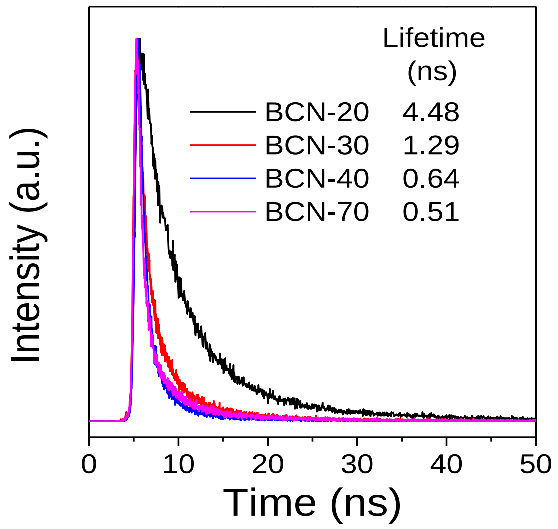

Supplementary Figure 11. Photoluminescence lifetime decay of the BCN-x samples.

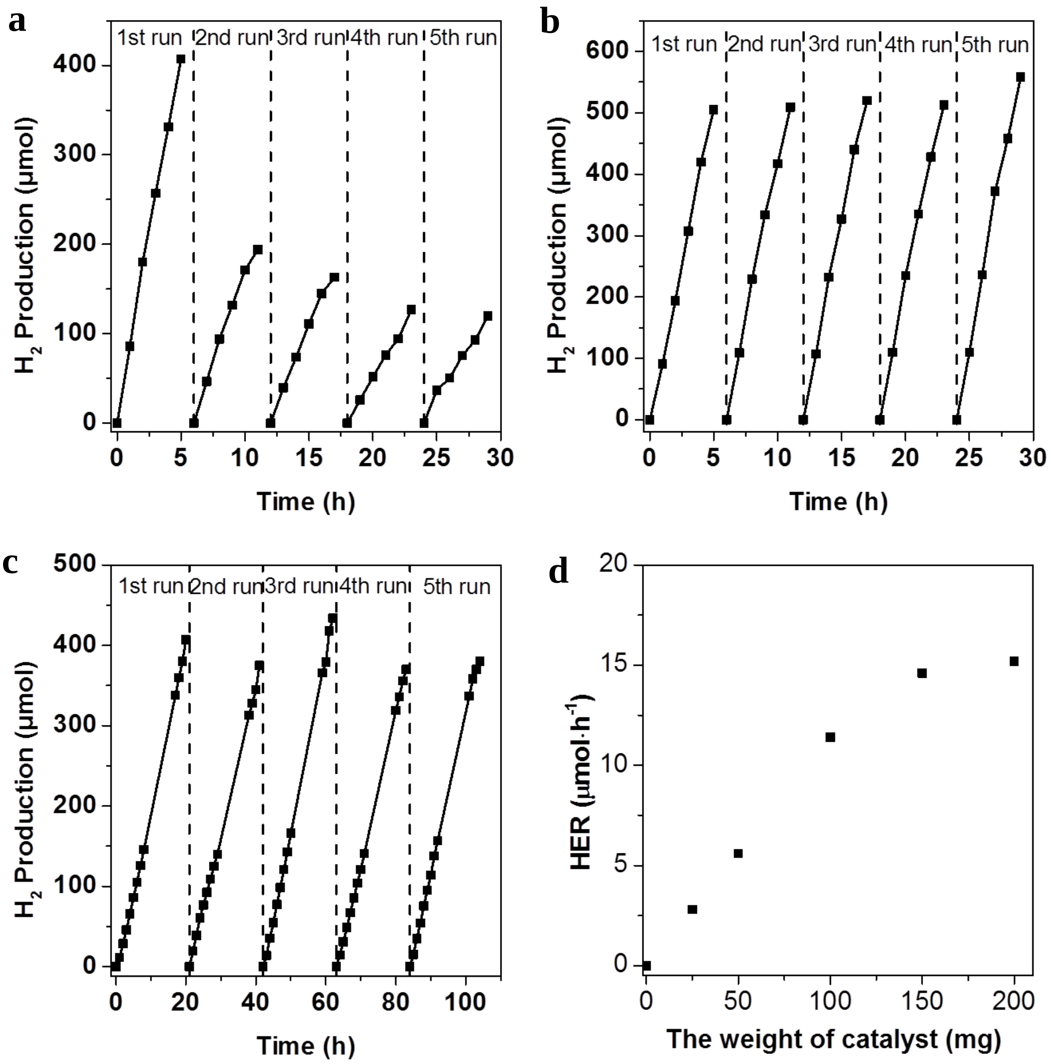

Supplementary Figure 12. Photocatalytic stability test. a, Stable hydrogen evolution from water by unmodified BCN-30 under ultraviolet light (λ> 300 nm) for 30 h. **b,** Stable hydrogen evolution from water by 1.0 wt% Pt-loaded BCN-30 under ultraviolet light for 30h. **c,** Stable hydrogen evolution from water by 1.0 wt% Pt-loaded BCN-30 under visible light (> 420 nm) for 104 h. The reaction was continued for many hours with evacuation each run (dashed line). **d,** The rate of hydrogen production over 1.0 wt% Pt-loaded BCN-30 with different amount of the catalyst with visible light illumination (> 420 nm). For these set of

experiments (a, b, and c), the amount of BCN-30 catalysts was 100 mg so that after the reaction we can recovered enough amount of the catalyst after the reaction for structural characterizations (XRD, FTIR and TEM).

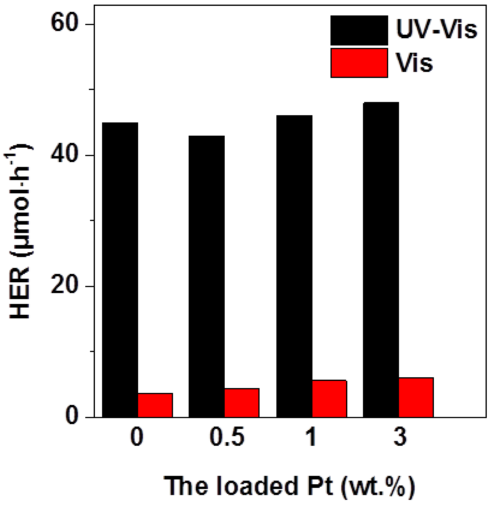

Supplementary Figure 13.. The rate of hydrogen production over BCN-30 loaded with different amount of Pt.

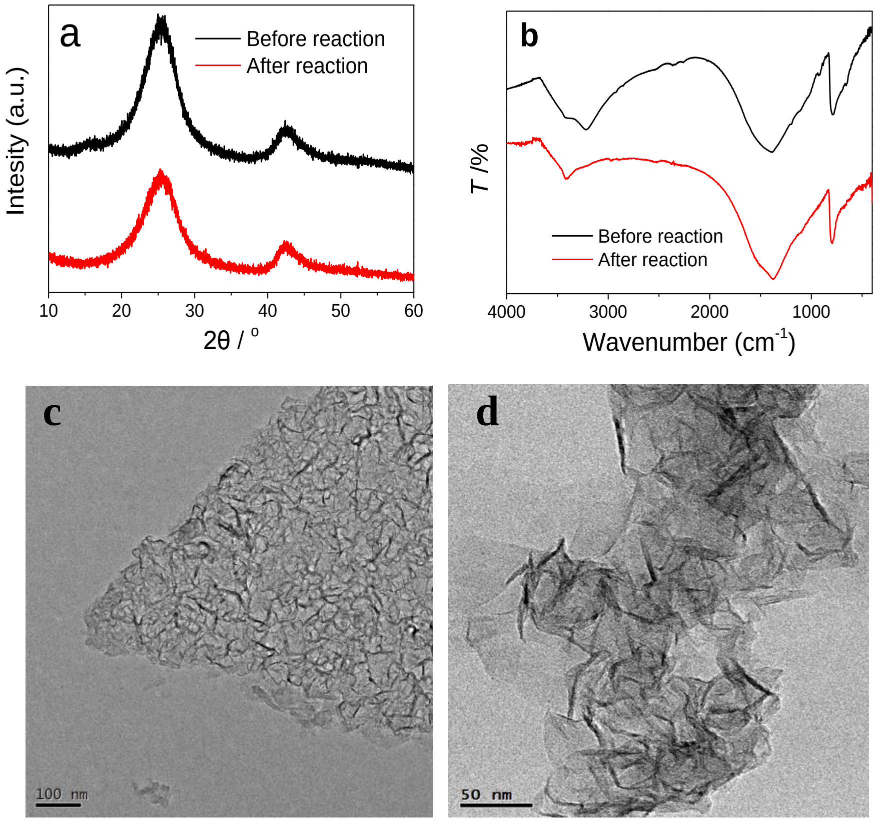

Before reaction After reaction

Supplementary Figure 14. XRD patterns (a), FT-IR spectra (b) and TEM (c,d) images of the BCN-30 sample before and after the photocatalytic hydrogen evolution reactions.

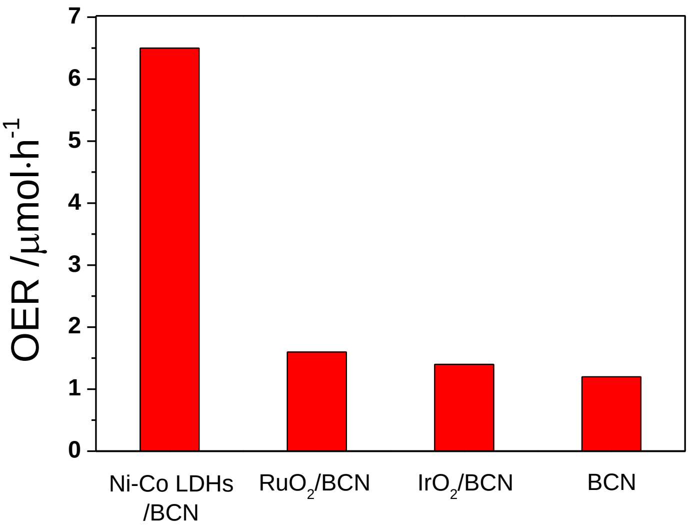

Supplementary Figure 15. The oxygen evolution rate (OER) over BCN-30 loaded with different co-catalysts under ultraviolet light irradiation (λ> 300 nm).

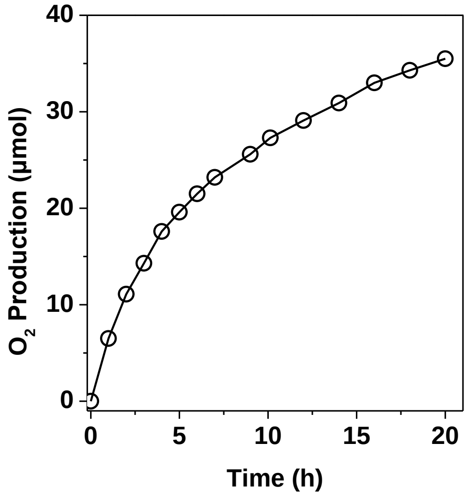

Supplementary Figure 16. Time course of photocatalytic O_2 production over Ni-Co LDHs/BCN-30 sample under ultraviolet light irradiation (λ> 300 nm).

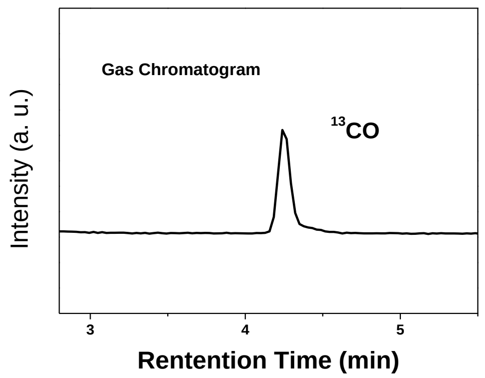

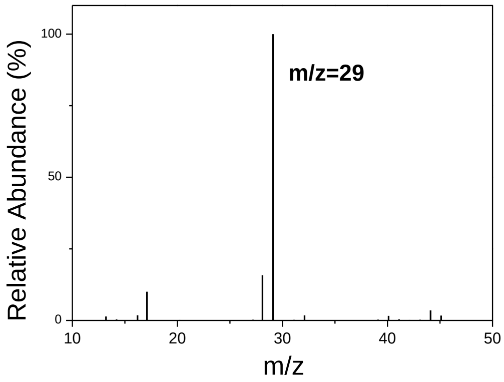

Supplementary Figure 17. Gas chromatogram (up) and mass spectra (m/z = 29) (down) analyses of the carbon source of the generated CO from the BCN-30 promoted photocatalytic $^{13}CO_2$ reduction system.

Supplementary Table 1. Elemental analysis results from XPS for BCN samples.

Sample	Boron (wt. %)	Carbon (wt. %)	Nitrogen (wt. %)	Oxygen (wt. %)
BCN-20	43.9	5.0	52.4	8.7
BCN-30	42.5	8.0	39.5	9.5
BCN-40	41.6	9.2	38.6	10.6
BCN-70	41.5	20.3	24.4	12.8

Supplementary Table 2. The apparent quantum efficiency (AQE) for photocatalytic hydrogen evolution over 1.0 wt% Pt-loaded BCN-x photocatalysts, using diode laser as the incident light (405 nm).

Sample	AQE (%)
BCN-20	0.05
BCN-30	0.54
BCN-40	0.44
BCN-70	0.08

Supplementary Table 3. The rate of hydrogen production over different photocatalysts under visible light (> 420 nm).

Sample	3 wt. % Pt	Without cocatalyst
BCN-30	6.0 μmol·h^{-1}	3.6 μmol·h^{-1}
TiO_2 (P25)	n.d. (no detected)	n.d.
g-C_3N_4	5.0 μmol·h^{-1}	0.1μmol·h^{-1}

Supplementary Table 4. The BET surface areas of the BCN-x and their photocataytic hydrogen evolution rate after loading with 1 wt% Pt as the cocatalysts**.**

Sample	HER[a] (μmol·h^{-1})	Specific surface area (m^2·g^{-1})	Pore Volume (cm^3·g^{-1})
BCN-20	0.8	488	0.24
BCN-30	5.6	520	0.26
BCN-40	2.7	559	0.28
BCN-70	1.0	680	0.34

There is not clear relationship between surface area and HER. This is due to the fact that in most solid-liquid phase heterogeneous photocatalysis the reaction rate is basically limited by charge separation instead of mass transfer as in the case of gas-solid phase heterogeneous photocatalysis.
